# Fructose drives mitochondrial metabolic reprogramming in podocytes via Hmgcs2-stimulated fatty acid degradation

**DOI:** 10.1038/s41392-021-00570-y

**Published:** 2021-07-09

**Authors:** Lei Fang, Tu-Shuai Li, Jing-Zi Zhang, Zhi-Hong Liu, Jie Yang, Bing-Hao Wang, Yu-Meng Wang, Jie Zhou, Ling-Dong Kong

**Affiliations:** 1 The State Key Laboratory of Pharmaceutical Biotechnology, Medical School, School of Life Sciences, Nanjing University, Nanjing, China; 2 Chemistry and Biomedicine Innovation Center of Nanjing University, Nanjing, China

**Keywords:** Cell biology, Kidney diseases, Predictive markers, Bioinformatics, Kidney diseases

**Dear Editor**,

The increasing consumption of dietary fructose has been proposed as a major contributor to metabolic syndrome, which promotes glomerular podocyte injury and proteinuria.^1^ Mitochondria are the key organelles for cellular bioenergetics to maintain energy homeostasis and gluconeogenesis.^2^ Mitochondrial damage may be linked to kidney diseases, in particular glomerular nephropathy.^3^ Our previous study showed that high fructose disturbed homeostasis of glycolipid metabolism in rat kidney and cultured differentiated podocytes.^4^ However, the mechanisms underlying high fructose-induced mitochondria dysfunction in glomerular podocyte injury are not well understood.

Here, we established a rat model fed with high fructose for 4, 8, 12, and 16 weeks (Supplementary Fig. S1) and used quantitative proteomic strategy to comprehensively characterize the dynamic changes in the progression of high fructose-induced glomerular podocyte injury of rats (Supplementary Fig. S2). Principal component analysis showed that N16 and M16 were deviated from the other six samples, which might be caused by the rat age increase and elder rat sensitivity in response to high fructose (Supplementary Fig. S2C). Dramatic mitochondria metabolic reprogramming including tricarboxylic acid cycle, fatty acid degradation, and oxidative phosphorylation driven by long-term high fructose feeding was observed (Fig. 1a and Supplementary Figs. S2e-l and S3–5). Mitochondrial swelling was detected from the fourth week of fructose modeling in rat glomerular podocytes compared with the corresponding normal group (Supplementary Fig. S6a). The decreased number of mitochondria as well as enlarged mitochondria with reduced matrix density and cristae numbers were further calculated in glomerular podocytes of rats at the 8th and 12th week of fructose modeling (Fig. 1b–d and Supplementary Fig. S6b, c). Moreover, at the 12th week, the ultrastructure of mitochondria was severely disrupted with cristae rupture and ablation in glomerular podocytes of high fructose-fed rats (Fig. 1b). Meanwhile, the mitochondria depolarization and ΔΨm reduction were detected in fructose-exposed differentiated podocytes (Fig. 1e and Supplementary Fig. S6d). In addition, high fructose exposure significantly decreased basal rate, ATP generation, and maximal respiration in differentiated podocytes starting from 48 h (Fig. 1f and Supplementary Fig. S6e–g), suggesting that high fructose induced mitochondrial dysfunction in podocytes.

**Fig. 1:**
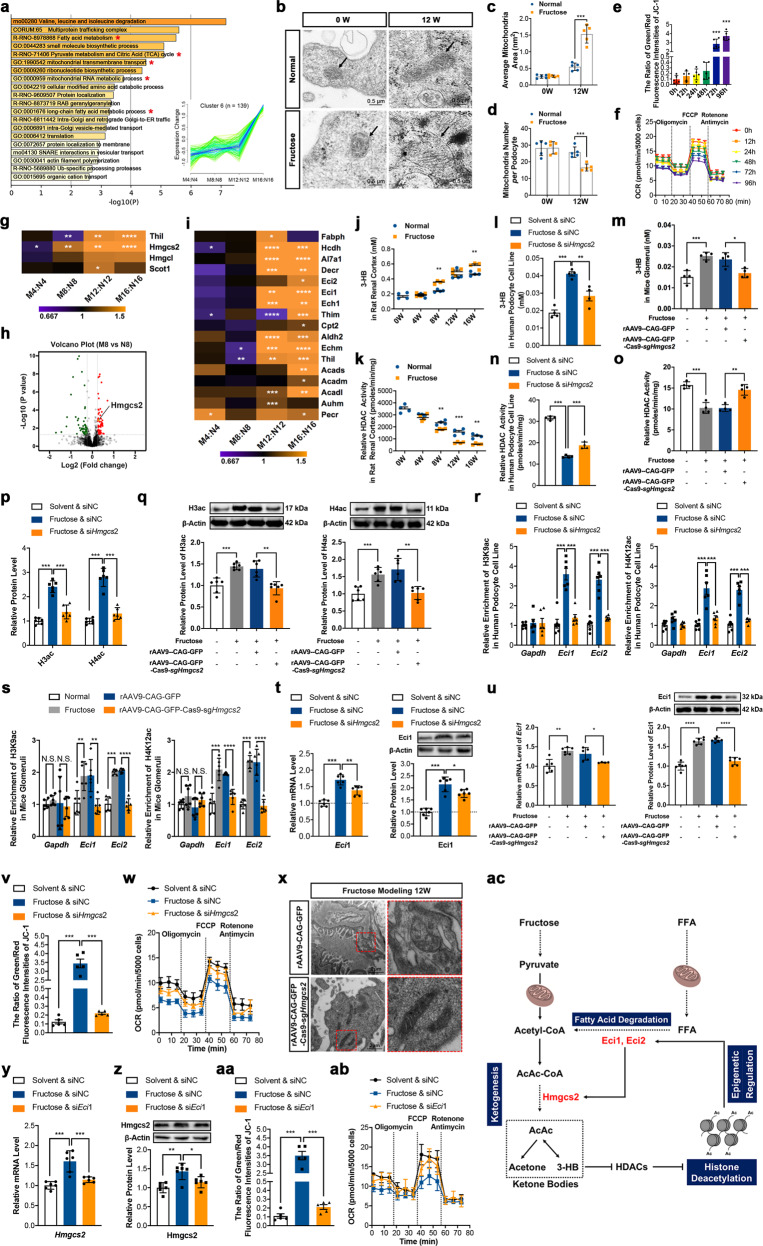
High fructose drives mitochondrial metabolic reprogramming in podocytes via Hmgcs2-stimulated fatty acid degradation. **a** Quantitative proteomics revealed a continuously upregulated protein cluster causing mitochondria-related events in rat glomeruli during 16-week fructose modeling. **b**–**d** The mitochondria ultrastructure was severely disrupted in glomerular podocytes of high fructose-fed rats. **b** Representative images of mitochondria ultrastructure by transmission electron microscopy. The size and total number of mitochondria in glomerular podocytes of normal and model groups are shown in **c**, **d**, respectively (*n* = 5 per group). **e** The mitochondria depolarization and ΔΨm reduction were detected in 5 mM fructose-exposed differentiated podocytes (*n* = 5 per group). **f** Mito stress test was performed to measure the key parameters of mitochondrial function in differentiated podocytes with or without 5 mM fructose exposure (*n* = 10 per group). The quantification of OCR data in **f**, **w**, **ab** have been normalized by mitochondrial protein. **g**, **h** Ketogenesis was significantly upregulated in rat glomeruli starting from the eighth week of fructose modeling. Upregulation of ketogenesis was shown by both heatmap (**g**) and volcano plot (**h**). **i** Fatty acid degradation was significantly upregulated in rat glomeruli starting from the 12th week of fructose modeling. **j** Time-dependent increase of ketone body 3-HB in rat renal cortex during fructose modeling (*n* = 4 per group). **k** HDACs activity was time-dependently inhibited in renal cortex of high fructose-fed rats (*n* = 4 per group). **l**, **m** Knockdown of *Hmgcs2* reversed upregulation of 3-HB induced by high fructose in differentiated podocytes (**l**) and in mouse glomeruli (**m**) (*n* = 4 per group). **n**, **o** Knockdown of *Hmgcs2* partially rescued the inhibited HDACs activity induced by high fructose in differentiated podocytes (**n**) and in mouse glomeruli (**o**) (*n* = 4 per group). **p**, **q** Knockdown of *Hmgcs2* reversed upregulation of H3ac and H4ac induced by high fructose in differentiated podocytes (**p**) and in mouse glomeruli (**q**) (*n* = 6 per group). **r**, **s** The increasing levels of H3K9ac and H4K12ac at *Eci1* or *Eci2* promoter induced by high fructose were significantly reversed by knockdown of *Hmgcs2* in differentiated podocytes (**r**) and mouse glomeruli (**s**). Enrichment of H3K9ac and H4K12ac at *Eci1* or *Eci2* promoter were measured by real-time qPCR and quantitated in differentiated podocytes or mouse glomeruli under indicated conditions. *Gapdh* was used as negative control (*n* = 6 per group). **t**, **u** Knockdown of *Hmgcs2* reversed the increase of Eci1 induced by high fructose in differentiated podocytes (**t**) and mouse glomeruli (**u**) as measured by real-time qPCR and Western blot (*n* = 6 per group). Relative mRNA or protein levels of Eci1 were normalized to β-Actin, respectively. **v** Knockdown of *Hmgcs2* efficiently rescued the decrease of mitochondrial membrane potential induced by high fructose in differentiated podocytes. Flow cytometric analysis of ΔΨm was performed in differentiated podocytes transfected with *Hmgcs2* siRNA as well as siNC and then cultured with or without 5 mM fructose (*n* = 5 per group). **w** Knockdown of *Hmgcs2* reversed the inhibitory effects of high fructose on basal rate, ATP generation, and maximal respiration in differentiated podocytes. Bioenergetics profile was measured by OCR with a Seahorse ×96 Extracellular Flux Analyzer (Seahorse Bioscience) in differentiated podocytes transfected with *Hmgcs2* siRNA as well as the respective negative control (siNC) and then cultured with or without 5 mM fructose (*n* = 6 per group). **x** Knockdown of *Hmgcs2* reversed the disruption of mitochondria ultrastructure observed by transmission electron microscopy in glomerular podocytes of high fructose-fed mice (*n* = 4 per group). **y**, **z** Knockdown of *Eci1* reversed the increase of Hmgcs2 induced by high fructose in differentiated podocytes. mRNA (**y**) and protein (**z**) levels of Hmgcs2 in differentiated podocytes transfected with *Eci1* siRNA as well as siNC and then cultured with or without 5 mM fructose (*n* = 6 per group). Relative mRNA or protein levels of Hmgcs2 were normalized to β-Actin, respectively. **aa** Knockdown of *Eci1* efficiently rescued the decrease of mitochondrial membrane potential induced by high fructose in differentiated podocytes. Flow cytometric analysis of ΔΨm was performed in differentiated podocytes transfected with *Eci1* siRNA as well as siNC and then cultured with or without 5 mM fructose (*n* = 5 per group). **ab** Knockdown of *Eci1* reversed the inhibitory effects of high fructose on basal rate, ATP generation, and maximal respiration in differentiated podocytes. Bioenergetics profile was measured by OCR with a Seahorse ×96 Extracellular Flux Analyzer (Seahorse Bioscience) in differentiated podocytes transfected with *Eci1* siRNA as well as the respective negative control (siNC) and then cultured with or without 5 mM fructose (*n* = 6 per group). **ac** Working model: an epigenetic modification mediated-positive feedback consisting of ketogenesis and fatty acid degradation drives mitochondrial metabolic reprogramming during fructose-induced podocyte injury. Mean values ± SD are shown. Student’s two-tailed paired *t* test was used for comparing two groups. One-way ANOVA with Tukey’s post hoc test was used for multi-group comparisons. *p < 0.05, **p < 0.01, and ***p < 0.001 denote the significant difference as compared to the corresponding normal animal group or normal cell group

In this study, high fructose feeding-developed rat model was quite different from diabetic kidney disease, because it did not cause high blood glucose even in long-term modeling of 16 weeks. During 16-week fructose modeling in rats, we observed continuously lipid accumulation in rat glomeruli starting as early as the fourth week (Fig. S1r, s). Importantly, 3-hydroxy-3-methylglutaryl-CoA synthase 2 (Hmgcs2), the core rate-limiting enzyme of ketogenesis, started to be significantly increased at the eighth week of fructose modeling in rat glomeruli compared with the corresponding normal group (Fig. 1g, h and Supplementary Figs. S4g–i and S7a). Interestingly, fatty acid degradation and fatty acid metabolism were downregulated at the 4th week, not significantly changed at the 8th week, but dramatically increased at both 12th and 16th weeks in rat glomeruli of fructose modeling vs. the corresponding normal group (Fig. 1i and Supplementary Figs. S2e–h and S7b). Upregulation of Hmgcs2 increased ketone bodies level, especially β-hydroxybutyrate (3-HB) (Fig. 1j and Supplementary Fig. S8a, b) to inhibit histone deacetylase (HDAC) activity in high fructose-fed rat glomeruli (Fig. 1k) and high fructose-exposed differentiated podocytes (Fig. S8c). Consequently, the overall acetylation of histone H3 and H4 was significantly upregulated in high fructose-exposed differentiated podocytes (Fig. 1p and Supplementary Fig. S8h) and glomeruli of high fructose-fed mice (Fig. 1q).

However, in *Hmgcs2* small interfering RNA (siRNA)-transfected differentiated podocytes and in the glomeruli of kidney-specific *Hmgcs2* knockdown mice, 3-HB level was reduced (Fig. 1l, m) and HDACs activity was significantly upregulated (Fig. 1n, o), whereas acetylation of histone H3 and H4 was significantly downregulated (Fig. 1p, q). Mechanically, histone H3 lysine 9 acetylation (H3K9ac), H3K14ac, H4K8ac, and H4K12ac at the promoter region of fatty acid degradation-related enzymes *Eci1* and *Eci2* were significantly enhanced in high fructose-exposed differentiated podocytes and glomeruli of high fructose-fed mice (Fig. 1r, s and Supplementary Figs. S8i, j and S9m, n). Downregulation of 3-HB production by transfecting *Hmgcs2* siRNA into high fructose-exposed differentiated podocytes or kidney-specific knockdown of *Hmgcs2* in mice prevented the increase of H3K9ac, H3K14ac, H4K8ac, and H4K12ac at *Eci1* and *Eci2* promoter regions (Fig. 1r, s and Supplementary Figs. S8i, j and S9m, n) and therefore reversed the upregulation of *Eci1* and *Eci2* at both the mRNA and protein levels induced by high fructose (Fig. 1t, u and Supplementary Figs. S8k, l and S9o, p). In addition, knockdown of *Hmgcs2* not only rescued the decrease of mitochondrial membrane potential (Fig. 1v and Supplementary Fig. S8q) as well as the inhibition of basal rate, ATP generation, and maximal respiration (Fig. 1w and Supplementary Fig. S8r–t) induced by high fructose in differentiated podocytes but also reversed the disruption of mitochondria ultrastructure in glomerular podocytes of high fructose-fed mice (Fig. 1x), suggesting an improvement of mitochondrial function. Taken together, these results suggest that Hmgcs2-controlled increase of 3-HB was responsible for the upregulation of fatty acid degradation-related enzymes and contributed to mitochondrial dysregulation in high fructose-induced podocyte injury.

Unexpectedly, we found that knockdown of *Eci1* or *Eci2* by RNA interference markedly inhibited the increase of Hmgcs2 induced by high fructose in differentiated podocytes (Fig. 1y, z and Supplementary Fig. S10e, f), indicating that the expression of key enzymes in ketogenesis may be regulated by fatty acid degradation pathway. More interestingly, downregulation of *Eci1* or *Eci2* efficiently rescued the decrease of mitochondrial membrane potential (Fig. 1aa and Supplementary Fig. S10a, g–h) and also the inhibitory effect on basal rate, ATP generation, and maximal respiration induced by high fructose in differentiated podocytes (Fig. 1ab and Supplementary Fig. S10b–d, i–l). Thus, our study reveals an epigenetic modification mediated-positive feedback mechanism containing ketogenesis and fatty acid degradation to regulate mitochondrial metabolic reprogramming during high fructose-induced podocyte injury (Fig. 1ac).

In conclusion, for the first time, we used systematic and high-throughput proteomic approach to comprehensively characterize the dynamic changes in glomeruli of high fructose-fed rats and revealed global fructose-driven mitochondria metabolic reprogramming. More importantly, this study provides new insights into the mechanism of high fructose-induced glomerular podocyte injury, demonstrating that the remodeling of metabolic pattern exacerbates podocyte injury via disturbing mitochondrial structure and function. Particularly, the enhancement of ketogenesis and fatty acid degradation pathways induced by high fructose participates in mitochondrial dysfunction ultimately leads to podocyte injury. This study is the first linking mitochondria metabolic reprogramming to podocyte injury induced by high fructose. Thus, inhibition of key enzymes in ketogenesis and fatty acid degradation efficiently prevents high fructose-induced mitochondrial dysfunction and therefore provides promising therapeutic targets for high fructose diet-induced podocyte injury.

## Supplementary information

Supplemental Material
