# Supplementary material for: Fructose drives mitochondrial metabolic reprogramming in podocytes via Hmgcs2-stimulated fatty acid degradation
Source: Signal Transduct Target Ther. 2021 Jul 9;6:253. doi: 10.1038/s41392-021-00570-y (PMC8266798; doi:10.1038/s41392-021-00570-y)
Supplement: Supplementary file 1 — Supplemental Material [file 41392_2021_570_MOESM1_ESM.docx]

**Supplemental Material for**

**Fructose Drives Mitochondrial Metabolic Reprogramming in Podocytes *via* Hmgcs2-stimulated Fatty Acid Degradation**

Lei Fang^1,2,§,*^, Tu-Shuai Li^1,§^, Jing-Zi Zhang^1,2,§^, Zhi-Hong Liu^1^, Jie Yang^1^, Bing-Hao Wang^1^, Yu-Meng Wang, Jie Zhou^1^, Ling-Dong Kong^1,*^

^1^The State Key Laboratory of Pharmaceutical Biotechnology, Medical School, School of Life Sciences, Nanjing University, Nanjing, 210023, China.

^2^Chemistry and Biomedicine Innovation Center of Nanjing University, Nanjing, 210023, China.

^§^: These authors contribute equally to this work.

^*^: Corresponding authors

^*^Correspondence should be addressed to Dr. Lei Fang (njfanglei@nju.edu.cn) and Dr. Ling-Dong Kong (kongld@nju.edu.cn), Nanjing University, Nanjing, 210023, People’s Republic of China. Phone: +86-25-83594691. Further information and requests for resources and reagents should be directed to and will be fulfilled by the Lead Contact, Dr. Ling-Dong Kong (kongld@nju.edu.cn).

**This file includes:**

Materials and Methods

References

Figure S1 to S10and Table S1 to S3

**Materials and Methods**

**Animals and high fructose diet modeling**

All animal experimental operations were approved by the Institutional Animal Care and Use Committee of Nanjing University. Male Sprague-Dawley rats (5 weeks old, 180-220 g) were obtained from Beijing Weitong Lihua Laboratory Animal Technology Co., Ltd (Beijing, China). The rats were housed with water and food *ad libitum* in a specific pathogen-free, temperature- and humidity-controlled environment (22 ± 2 °C, 50 ± 5% humidity) with a normal 12h light/dark cycle. Seventy-two rats were grown to 5 weeks, and divided into two experimental groups: normal group (N, n=40) with a standard chow and water (available *ad libitum*) and high fructose-treated group (M, n=32) with a standard chow and drinking water containing 10% fructose solution (W/V, available *ad libitum*) for 4, 8, 12 and 16 weeks, respectively. Body weight was measured once a week throughout animal experiment. Once every 4 weeks, eight rats were randomly selected from normal group and fructose-fed group. Each rat was fed in a metabolic cage to obtain urine for 24 h. After centrifugation for 10 min at 3,000 *g*, 4 °C, urine was obtained and then stored at -80 °C for further analysis. In this study, we tried our best to reduce the number of rats used, and to minimize animal suffering.

**Generation of kidney-specific *Hmgcs2* knockdown (KD) mice**

C57BL/6N mice aged 4-5 weeks were purchased from Beijing Weitong Lihua Laboratory Animal Technology Co., Ltd. (Beijing, China; production license: SCXK 2016-0006) and bred continuously in a specific pathogen-free environment with temperature- and humidity-controlled (22 ± 2 °C, 50 ± 5% humidity). All mouse procedures were performed according to the regulations of the Institutional Animal Care and Use Committee of Nanjing University.

For kidney specific knockdown of *Hmgcs2*, *Hmgcs2* specific sgRNA (5’- GGACTTGTCAATGATGGTCT-3’) and negative control (NC) sgRNA (5’-AATCAACCGTGATAGTCTCG-3’) were designed and cloned into pLV-U6-sgRNA vector which co-expresses hCas9. The corresponding rAAV9-CAG-GFP-vehicle and rAAV9-CAG-GFP-Cas9-sg*Hmgcs2* were generated, and an optimized kidney-targeted gene delivery strategy was adopted with rAAV9 administered *via* retrograde renal vein injection in mice. Detailed operation process was performed as elaborated previously^1^. Briefly, after mice anesthetization, kidneys were exposed through the flank incision. Then, rAAV9 particles were inoculated into a vein using a 31G needle while the vein was clamped. Next, the clamp was taken out after 15 min post-injection and the incision was sutured. After two weeks post-injection, epifluorescent microscopy, real-time quantitative PCR (real-time qPCR) and Western blot analysis were used to detect the *Hmgcs2* KO efficiency of rAAV9 in mouse kidney. Mice injected with rAAV9-CAG-GFP-vehicle or rAAV9-CAG-GFP-Cas9-sg*Hmgcs2* were then fed with 30% fructose solution in drinking water (wt/vol, available *ad libitum*) for another 12 weeks. Mice with standard diet and drinking water (available *ad libitum*) or standard diet and 30% fructose solution in drinking water (wt/vol, available *ad libitum*) were used as normal and model control, respectively. At the 11^th^ week of fructose modeling, the mice were placed in metabolic cage to collect 24 h urine for the detection of urine protein and creatinine. We have tried our best to reduce animal suffering and the number of animals used.

**Reagents and antibodies**

For animal experiments, fructose was provided by Shandong Xiwang Sugar Industry Co., Ltd. (Binzhou, China). For cell experiments, fructose and RPMI-1640 were purchased from Tongying Biotechnology Co., Ltd. (Nantong, China). Alexa Fluor 488 goat anti-rabbit IgG (0423), Trizol reagent and Lipofectamine 2000 were from Invitrogen Biotechnology Company (Shanghai, China). Rat albumin ELISA kit was from Excell Bio Corporation (Wellington, New Zealand). Reverse transcription system kit, MultiScribe reverse transcriptase, SYBR^®^ Green Supermix, and trypsin were purchased from Bio-Rad Inc. (California, USA). Mitochondrial membrane potential assay kit, 4, 6-diamidino-2-phenylindole (DAPI) staining solution and cell lysis RIPA buffer were from Beyotime Biotechnology (Nanjing, China). The assay kits for uric acid, urine protein, creatinine, total cholesterol test kit and triglyceride were from Qixiang Biotechnology Co., Ltd (Weifang, China). HDACs enzyme activity detection kit, acetoacetate (AcAc) colorimetric assay kit, acetone ELISA detection kit and 3-HB (ketone body) colorimetric assay kit were purchased from Active Motif, Biovision (Shanghai, China), Keshun Biotechnology (Shanghai, China), and Cayman Biotechnology Co., Ltd (Shanghai, China), respectively. Nuclear Protein Extraction kit was purchased from Sigma-Aldrich Inc. (St. Louis, MO). Recombinant Human IFN-gamma Protein was purchased from R&D Systems (Minneapolis, USA). HRP-conjugated mouse anti-IgG (ab19195), HRP-conjugated goat anti-IgG (ab6656), Rabbit anti-H3K9ac (ab4441), anti-H3K14ac (ab52946), anti-H4K8ac (ab45166), anti-H4K12ac (ab46983), rabbit anti-Histone H3 (ab1791), rabbit anti-Histone H4 (ab10158), rabbit anti-Aquaporin 1 (ab65837) and rabbit anti-Lamin A antibody (ab26300) were from Abcam (Cambridge, UK). Rabbit anti-Eci1 (11535-1-AP) and anti-CD31 (11265-1-AP) were from Proteintech Corporation (Chicago, USA). Mouse anti-synaptopodin (MAB4919) was from Abnova (Tokyo, Japan). BCA protein assay kit, mouse anti-Eci2 (MA5-25865), rabbit anti-Hmgcl (PA5-21996), 4,4-difluoro-1,3,5,7,8-pentamethyl-4-bora-3a, and 4a-diaza-s-indacene (BODIPY^®^493/503) were purchased from ThermoFisher (Waltham, USA). Rabbit anti-Hmgcs2 (MBS7106943) and rabbit anti-Histone H1.4 (MBS1750286) were purchased from MyBioSource Co., Ltd (San Diego, USA). Goat anti-WT1 (sc-15421) was purchased from Santa Cruz (Santa Cruz, USA). Mouse anti-β-Actin (ABM-0001) was from Zoonbio Biotechnology Company (Nanjing, China). Rabbit HRP-conjugated rabbit anti-IgG (#AP132P) and SimpleChIP^®^ Enzymatic Chromatin Immunoprecipitation Kit were obtained from Cell Signaling Technology (Cambridge, USA).

**Blood and tissue sample preparation**

Once every 4 weeks, eight rats which were randomly selected from the normal group and fructose-fed group were anesthetized intraperitoneally with sodium pentobarbital (50 mg/kg; intraperitoneal injection). Blood were collected from rat abdominal artery followed by centrifugation at 3,000 g for 15 min to obtain the serum. Then, rat kidney cortex tissues were processed into equal pieces for glomeruli isolation, as well as for histology, electron microscopy analysis and total RNA isolation, respectively. The graded sieving technique was used to isolate rat glomeruli using steel sieves with pore sizes of 250, 150 and 75 μm. 4% paraformaldehyde was used to fix the rat kidney cortex tissues forhematoxylin-eosin (HE) staining, periodic acid-Schiff (PAS) staining and Masson staining. The serum, rat kidney cortex tissues and glomerulus samples were then kept at -80 °C for further experiments.

**Biochemical analysis**

The levels of serum uric acid were tested using the phosphotungstic acid method. The concentrations of urinary albumin were detected using a rat albumin ELISA kit and showed as the value corrected according to urinary creatinine level. The levels of urea nitrogen and creatinine in serum were measured using commercially biochemical detection kits, respectively. All operations were strictly in accordance with the manufacturer's requirements.

**Histologic analysis**

The fixed renal tissue was embedded in paraffin and then cut into sections (4 μm). After deparaffinization, HE staining, PAS staining and Masson staining were carried out. Changes in eight randomly selected glomeruli were observed using a light microscope (× 500).Frozen sections were prepared for Oil Red O staining to evaluate glomerular accumulation of neutral fats. The stained kidney sections were imaged on the Olympus microscope and quantified *via* densitometry using ImageJ (version 1.42q, National Institutes of Health).

**Lipid droplet determination by flow cytometry**

Flow cytometry was used to quantify lipid droplets accumulation in the rat renal cortex. Single-cell suspension of rat renal cortical tissue was prepared by collagenase (Shanghai Tuoyang Biotechnology; Y020055) digestion. Renal cortical tissue was minced with sterile scissors, and incubated with 1 mL of the collagenase solution (0.5 mg/mL) at 37 °C for 40 min. The cells were collected by the centrifugation at 500 *g* for 15min at 4 °C. Re-suspend the cells in the cold PBS and centrifuge 1,000 *g* for 5 min. Cells were re-suspended in the PBS, and counted with the use of a hemocytometer. A total of 5 × 10^5^ cells were treated with 2 μg/mL BODIPY® Lipid Probes 500/510 (4,4-difluoro-3a,4adiaza-s-indacene; Invitrogen) for 30 min at room temperature in the dark. Lipid droplet was determined by FACScalibur flow cytometer (BD Biosciences, Franklin Lakes, USA) for measurement of the mean fluorescence intensity.

**Immunofluorescence**

Rat renal cortex samples were fixed with 4% paraformaldehyde and embedded in paraffin. After antigen retrieval, paraffin sections (5 μm) were blocked with 10% bovine serum albumin (BSA) for 1 hour at room temperature. Primary antibodies were diluted in PBS and incubated on the sections overnight at 4 °C. To visualize primary antibodies, secondary antibodies goat anti-rabbit/FITC (Hmgcs2, Eci1, H3K9ac, H3K14ac, H4K8ac, H4K12ac, Aquaporin 1), goat anti-mouse/Cy3 (CD31, Synaptopodin), donkey anti-goat/FITC (WT1) were used for 50 min at room temperature. Nuclei were counterstained with DAPI for 10 min (Servicebio, G1012). Fluorescent images were acquired by fluorescence microscopy (NIKON ECLIPSE C1) at × 400 magnification.

**Transmission Electron Microscope (TEM)**

For TEM, several 1mm cubes from rat kidney cortex were cut and fixed in 2.5% glutaraldehyde for 5 h. After being washed in cacodylate buffer, the cubes werepost-fixed in 1% osmium tetroxide, and then block-stained with uranyl acetate before embedding in Poly/Bed812 resin. Finally, the thin sections were stained with uranyl acetate and lead citrate, and observed in a JEOL-JEM 1010 transmission electron microscope (JEOL, Tokyo, Japan) at an accelerating voltage of 80 kV. Digital images were collected using the AMT Imaging System (Advanced Microscopy Techniques Corp, Danvers, MA).

**Protein extraction, digestion and labeling with iTRAQ reagents**

100 mg of rat glomerulus sample was used for protein extraction. Briefly, the rat glomeruli were first homogenized in RIPA lysis buffer, incubated for 30 min on ice, then followed by non-contact ultrasonic decomposition at 4 °C using Bioruptor Plus sonication device (Diagenode, Belgium). The lysates were centrifuged at 12,000 rpm for 30 min to collect the supernatant. The protein concentration was determined with the Pierce™ BCA protein assay kit and further confirmed by Coomassie brilliant blue staining.

The on-filter protein digestion and iTRAQ labeling were performed as previously described^2^. After the trypsin digestion, the resultant peptides were collected and labeled with iTRAQ Reagent 8-plex multiplex kit (SCIEX) according to the manufacturer’s instructions. The samples were labeled as shown in Table S1. In one individual iTRAQ experiment, four glomerulus samples from normal rats at 4^th^, 8^th^, 12^th^, and 16^th^ week (N4, N8, N12 and N16) were labeled with iTRAQ tag 113, 115, 117 and 119, respectively. And four glomerulus samples from fructose modeling rats at 4^th^, 8^th^, 12^th^, and 16^th^ week (M4, M8, M12 and M16) were labeled with iTRAQ tag 114, 116, 118 and 121, respectively. Two independent biological replicates were set. For each iTRAQ experiment, all labeled peptides were finally separated into 16 samples for LC-MS/MS analysis.

**LC-MS/MS and database searching**

MS data acquisition was performed with a NanoLC.2D (Eksigent Technologies) coupled with a TripleTOF 5600^+^ System (SCIEX) as previously described^2^. The original MS/MS data were submitted to ProteinPilot Software (version 4.5, AB Sciex) for data analysis and searched against *Rattus norvegicus* in UniProt database concatenated with reverse decoy database (April 9, 2019, containing 36,141 sequences, http://www.uniprot.org/proteomes/UP000002494). The searching parameters were set same as previously used.

**Bioinformatics analysis**

Further bioinformatics analyses were carried out with R studio and other requisite software. For overview of mass spectrometry data, principal component analysis (PCA) (the function “prcomp” from the R package “stats”), whole-proteome heatmap (the R package “pheatmap”) and hierarchical cluster analysis (the R package “hclust”) were performed to demonstrate the similarity or heterogeneity in all eight “N” and “M” samples in the same iTRAQ experiment. For quality control of biological replicates, the number of identified and quantified proteins were calculated for each iTRAQ experiment; venn diagram (the R package “VennDiagram”) and heatmap (the R package “pheatmap”) have shown high reproducibility between two biological replicates.

Moreover, the overall dynamic changes of protein expression between “N” and “M” samples from the same time point were displayed using volcano plot with the R package “ggplot2” (the cutoff value of statistical significance was *p* < 0.05, fold change was ≥ 1.2 or ≤0.83). And the reproducibility of potential differentially expressed proteins across different time points was performed by the R package “VennDiagram”.

For in-depth information, the stable differentially expressed proteins in M vs. N were classified by GO and KEGG pathway annotation and enrichment using DAVID online tools (http://david.abcc.ncifcrf.gov). The GO or KEGG pathway with a corrected *p* value < 0.05 was considered as significant. For each category, a two-tailed Fisher’s exact test was employed to test the enrichment of the differentially expressed protein against all identified proteins (Prism 8 software). For each KEGG category related with mitochondrial metabolism, heatmap of M vs. N matrix was performed to show the trend of concerned metabolic events accompanied with fructose modeling at protein level, and KEGG pathway mapping (https://www.kegg.jp) was used to map the distribution and coverage of differentially expressed protein at all four time points on concerned metabolic pathways. Then, the trend of M vs. N matrix was soft clustered by the R package “Mfuzz”, and the clusters continuously increasing or decreasing were selected for further Protein-Protein Interaction analysis, Gene Ontology (GO) and Kyoto Encyclopedia of Genes and Genomes (KEGG) pathway annotation clustering using Metascape (http://metascape.org).

**Cell culture and treatment**

Human podocytes cell line (passage 10-25) was got from Dr. Zhi-Hong Liu (National Clinical Research Center of Kidney Disease, Jinling Hospital, Nanjing, China), which was originally obtained from M. Saleem (University of Bristol, Bristol, United Kingdom).The podocytes were cultured as previously described^3^. Differentiated podocytes were treated with or without 5 mM fructose for 0, 12, 24, 48, 72 or 96 h to detect protein and mRNA levels of Eci1, Eci2, Hmgcl and Hmgcs2, mitochondrial transmembrane potential (*△Ψ*m), oxygen consumption rate (OCR), and HDACs activity, as well as ketone bodies AcAc, 3-HB and acetone levels, respectively.

siRNA of *Eci1*, *Eci2* or *Hmgcs2* as well as their corresponding normal controls were transiently transfected into differentiated podocytes using Lipofectamine 2000, according to the instructions provided by the manufacturer, respectively. The siRNA nucleotide sequences were displayed in Table S2. The efficiency of siRNA was measured by detecting *Eci1*, *Eci2* or *Hmgcs2* mRNA levels at 24 h using real-time qPCR, and Eci1, Eci2 or Hmgcs2 protein levels at 48 h by Western blot, respectively. After 6 h, *Eci1*or *Eci2* siRNA-transfected differentiated podocytes were then cultured in RPMI-1640 medium containing 10% FBS with 5 mM fructose for 72 h to test protein and mRNA levels of Hmgcl and Hmgcs2, △*Ψ*m, OCR, and HDACs activity as well as ketone bodies AcAc, 3-HB, and acetone levels, respectively. In all experiments, total cellular proteins were isolated. BCA protein assay kit was used to measure podocyte protein concentration. Each of cell experiment was repeated at least four times and all protein samples were kept at -80 °C before Western blot and real-time qPCR assay.

**Evaluation of mitochondrial membrane potential**

The △*Ψ*m of podocytes was detected using a JC-1 (5,5,6,6-tetrachloro-1,1,3,3-tetraethylbenzimidazolylcarbocyanine iodide) mitochondrial membrane potential assay kit according to the instruction of the manufacture. Briefly, the podocyte samples were washed using 100mM cold PBS four times after the digestion with trypsin without EDTA. The podocytes were re-suspended in RPMI-1640 medium and adjusted to 4 × 10^6^ cells/mL. Then, the podocytes were incubated at 37 °C for 20 min with 5 μg/mL JC-1, washed three times with JC-1 staining buffer and re-suspended in a proper amount of JC-1 staining buffer. Finally, the samples were measured using a FACSCalibur cytometer (Becton Dickinson) equipped with a 488 nm argon laser.

**Seahorse metabolic analyzer assays**

Differentiated podocytes were cultured in 6-well culture plates at a density of 5 × 10^4^ cells/mL with or without 5 mM fructose conditions for 0, 12, 24, 48, 72, or 96 h to detect OCR. Podocytes were then passaged to a 96-well XF96 Plate (Seahorse Bioscience, Billerica, MA) at a cell density of 5 × 10^4^ cells *per* well. Cartridge plates used for metabolic stress injection were hydrated for 14 h at 37 °C before the assay with seahorse XF calibrant (Seahorse Biosciences). One hour before the seahorse analysis, seahorse assay medium (final volume of 180 μL *per* well) was used to replace the XF96 plate running medium. The experimental conditions and operating procedures were carried out in accordance with the instructions of Seahorse Biosciences, respectively. OCR was reported in the unit of picomoles *per* minute, and the detected values were normalized to cell number with the BCA protein assay kit. The area under the curve represents the OCR value was automatically measured by Seahorse XFp software version 2.2.0 as previously reported^4^.

**Real-time quantitative PCR analysis**

Total RNA was extracted from rat glomeruli and differentiated podocytes with Trizol reagent according to the manufacturer’s requirements, respectively. For real-time qPCR analysis, RNA (1 μg) was reverse transcribed into single-stranded cDNA using oligo(dT) primers. The real-time qPCR analysis was carried out in triplicate with gene-specific primer sets and iTaqTM Universal SYBR^®^ Green Supermix. The sequence of primers used to amplify *Eci1*, *Eci2*, *Acca2* or *Hmgcs2* were listed in Table S3. All of primers were synthesized by GENEray Biotechnology (Shanghai, China). The real-time qPCR cycling conditions were 95 °C for 30 s followed by 40 cycles of 94 °C for 5 s and 60 °C for 30 s. ^△△^Ct statistical method was used to calculate relative expression of target genes under different experimental conditions with *β-Actin*, *Lamin A* or glyceraldehyde-3-phosphate dehydrogenase (*Gapdh*) serving as the reference gene. The expression of target gene was normalized to *β-Actin*, *Lamin A* or *Gapdh*, respectively.

**Western blot analysis**

For glomerular protein extract, rat glomeruli samples were homogenized in lysis buffer (10 mM Tris-HCl, 1 mM EDTA, and 250 mM sucrose, pH 7.4, containing 15 µg/mL aprotinin, 5 g/mL leupeptin, 0.1 mM PMSF, 1 mM NaF, and 1 mM Na_3_VO_4_) plus protease inhibitors and centrifuge at 12,000 *g* for 20 min at 4 °C. For the differentiated podocyte protein extract, after the preparation in the cell lysis buffer containing 0.1 mM PMSF, cell lysates were ultracentrifuged at 12,000 *g* for 5 min at 4 °C.

10% SDS-PAGE was used to analyze Eci1, Eci2, Hmgcl, Hmgcs2, Histone H1.4, H3ac and H4ac expression. The samples were blotted onto polyvinylidene difluoride membranes and then blocked in 5% non-fat dry milk in Tris-buffered saline (TBS) containing 0.1% Tween-20 for 1 h at room temperature. Then, the polyvinylidene difluoride membranes were incubated at 4 °C overnight with the following antibodies: rabbit anti-Eci1 (dilution 1:1,000), rabbit anti-Histone H1.4 (dilution 1:2,000), rabbit anti-Hmgcl (dilution 1:1,000), rabbit anti-Hmgcs2 (dilution 1:2,000), rabbit anti-Lamin A, rabbit anti-β-Actin (dilution 1:5,000) and mouse anti-Eci2 (dilution 1:2,000). Blots were then washed 5 times in TBS containing 0.1% Tween-20 for thirty minutes, incubated in HRP conjugated anti-rabbit or anti-mouse diluted (1:8000) in 5% milk in TBS containing 0.1% Tween-20 for one hour at 4 °C. Immunoreactive bands were visualized using enhanced chemiluminescent substrate and analyzed *via* densitometry through ImageJ software (version 1.42q, National Institutes of Health).

**HDACs activity assay**

Nuclear extract from the glomeruli of high fructose-fed and corresponding normal control rats or the differentiated podocytes was tested using commercial HDACs assay kit. All operations were in accordance with the manufacturer’s instructions. Briefly, after mixing the detection reagents with protein samples in NUNC Maxisorp 96-well antigen coated plates, wells were incubated at 37 °C for 30 min, and then added 0.8 M H_2_SO_4_ to stop the reaction. Finally, absorbance was recorded at 460 nm to evaluate HDACs activity using spectrophotometer (Eppendorf).

**Ketone body detection**

Renal cortex or differentiated podocytes were lysed with RIPA buffer and the lysates were centrifuged at 1,500 *g* for 15 min. β-Hydroxyl-butyrate levels were measured using β-hydroxyl-butyrate colorimetric assay kit. AcAc and acetone levels were quantified using AcAc colorimetric assay kit and acetone ELISA detection kit, respectively. All operating procedures were strictly performed according to the manufacturer's protocol.

**Chromatin Immunoprecipitation (ChIP)**

After transfection with *Hmgcs2* siRNA into 5 mM fructose-exposed differentiated podocytes for 72 h, *Hmgcs2* siRNA-transfected podocytes and their control were digested with trypsin and washed three times with PBS. These podocytes were then re-suspended using RPMI-1640 containing 1% formaldehyde for 10 min followed by the addition of glycine (adjust the final concentration to 0.125 M) at 25 °C for another 10 min. After homogenizing the podocyte pellet with PBS, 1 mL of 1% sodium dodecyl sulfate (SDS) lysis buffer was added. The podocyte samples were processed by ultrasonic cell disruption (25 cycles, every cycle: 35 s ON /40 s OFF) to generate 180-450 nucleotide fragments, and then centrifuged at 15,000 *g* at 4 °C. Supernatant samples were diluted with specific buffer (16.7 mM Tris-HCl, 1.1% Triton X-100, 167 mM NaCl, 1.2 mM EDTA), precleared with protein-G beads and then blocked with 1% BSA and salmon sperm DNA for 2 h. Next, 1 mg of H3K9ac, H3K14ac, H4K8 and H4K12ac antibodies were used to incubate with the precleared supernatant overnight at 4 °C, respectively. Immunoprecipitation with normal IgG matching the specific-antibody isotype was also performed as negative control. Then, the protein-G sepharose beads (Sigma-Aldrich Cat. No. P3296) were used to incubate with the supernatant samples for 2 h at 4 °C before centrifugation. After successively recovered and washed in low salt buffer, high salt buffer and LiCl buffer, the beads were washed in Tris-EDTA (TE) buffer for three times. Next, the washed beads was mixed with elution buffer (10 mM Tris-HCl, 0.5% SDS, 300 mM NaCl and 5 mM EDTA), and incubated with RNase at 37 °C for 1.5 h. Then, Proteinase K was added to the mixture at 65 °C overnight. The samples were mixed with equal amount of Phenol-Chloroform-Isoamyl alcohol before recovering the water phase. 100% ethanol, NaOAc and glycogen were used to precipitate DNA, and the sample was washed with 70% ethanol after centrifugation at 14,000*g* for 30 min at 4 °C. Finally, real-time qPCR was carried out in triplicate with SYBR Green I dye on the Real-Time PCR Detection System (Bio-Rad).

**Quantification and statistical analysis**

All data are presented as mean ± SD. Statistical analysis was performed using Prism (GraphPad). Paired or unpaired two-tailed Student’s *t* tests were used for comparing two groups. A two-tailed Pearson’s correlation test was used for correlation analysis. One-way ANOVA with Tukey’s post hoc test was used for multi-group comparisons. The single symbol ^*^ represent a *p* value of <0.05, two symbols ^**^ represent a *p* value of <0.01, and three symbols ^***^ denote a *p* value of <0.001 throughout the study.

**Data and software availability**

The mass spectrometry proteomics data have been deposited to the ProteomeXchange Consortium *via* the PRIDE^5^ partner repository with the dataset identifier PXD017849. Submission details: Project Name: High Fructose-Driven Epigenetic and Metabolic Reprogramming in Glomerular Podocyte. Project accession: PXD017849. Project DOI: Not applicable. Reviewer account details: Username: reviewer34542@ebi.ac.uk; Password: icXtyKXv.

**References**

1. Rocca, C.J., Ur, S.N., Harrison, F. & Cherqui, S. rAAV9 combined with renal vein injection is optimal for kidney-targeted gene delivery: conclusion of a comparative study. *Gene Ther***21**, 618-628 (2014).

2. Cao, Y.*, et al.* Significant Down-Regulation of Urea Cycle Generates Clinically Relevant Proteomic Signature in Hepatocellular Carcinoma Patients with Macrovascular Invasion. *J Proteome Res***18**, 2032-2044 (2019).

3. Wu, J.*, et al.* MicroRNA-30 family members regulate calcium/calcineurin signaling in podocytes. *J Clin Invest***125**, 4091-4106 (2015).

4. Lee, K.Y., Gesta, S., Boucher, J., Wang, X.L. & Kahn, C.R. The differential role of Hif1β/Arnt and the hypoxic response in adipose function, fibrosis, and inflammation. *Cell Metab***14**, 491-503 (2011).

5. Deutsch, E.W.*, et al.* The ProteomeXchange consortium in 2020: enabling 'big data' approaches in proteomics. *Nucleic Acids Res***48**, D1145-d1152 (2020).

**
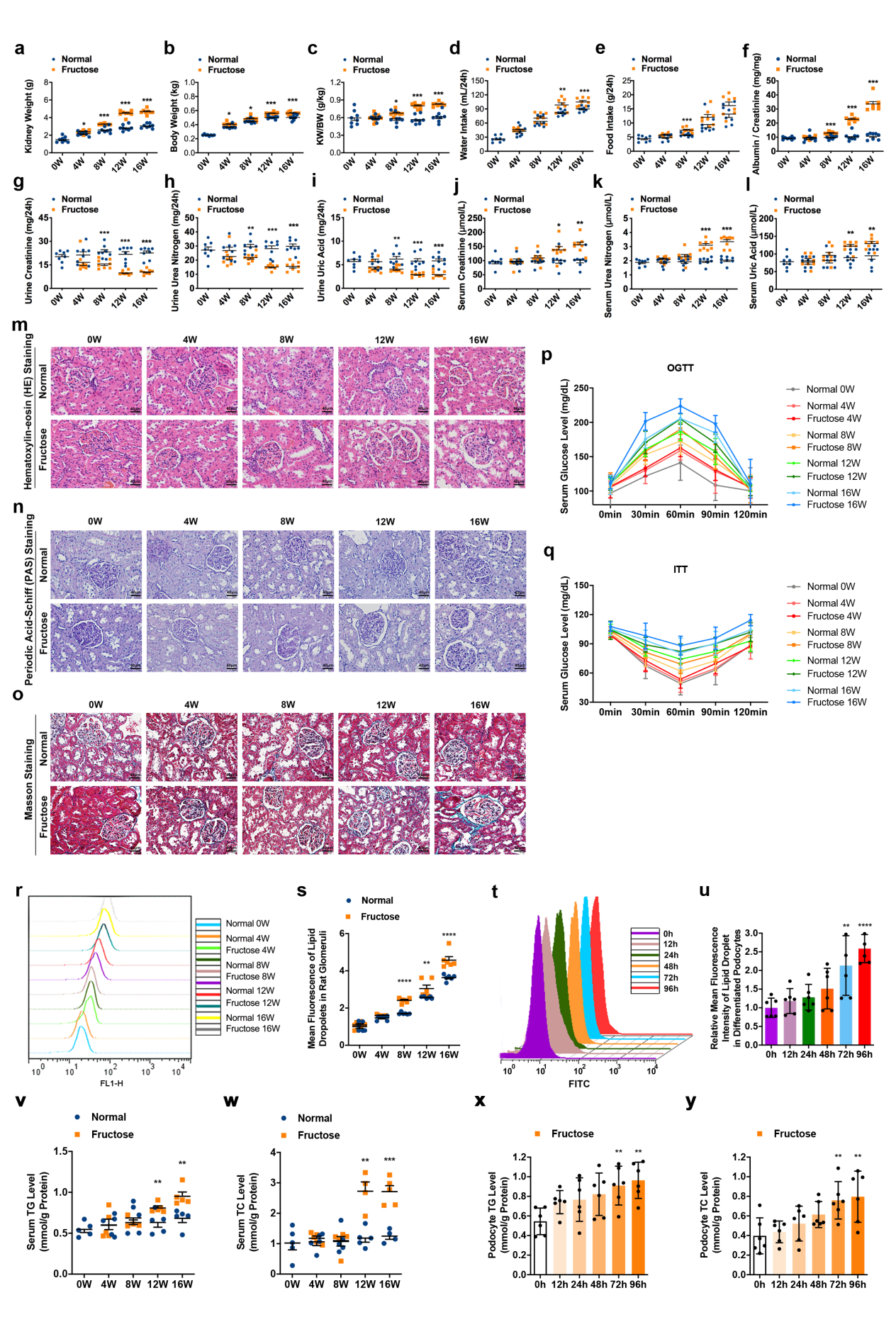
 Figure S1. Phenotypes related to metabolic disorder in high fructose-fed rats**
(a-c) Kidney weight (KW, a), body weight (BW, b) and KW to BW ratio (KW/BW, c) in high fructose-fed rats compared to normal rats.
(d-e) Metabolic cage to detect water (d) and food (e) intake in high fructose-fed rats compared to normal rats.
(f) Albumin to creatinine ratio in high fructose-fed rats compared to normal rats.
(g-i) Creatinine (g), urea nitrogen (h) and uric acid (i) levels in urine in high fructose-fed rats compared to normal rats.
(j-l) Creatinine (j), urea nitrogen (k) and uric acid (l) levels in serum in high fructose-fed rats compared to normal rats.
(m-o) Hematoxylin-eosin (HE) staining (m), Periodic acid-Schiff (PAS) staining (n) and Masson staining (o) of glomeruli in high fructose-fed and control rats at 0, 4, 8, 12 and 16 weeks, respectively.
(p-q) *In vivo* analysis of glucose and lipid metabolism in high fructose-fed rats using the oral glucose tolerance test (OGTT) (p) and insulin tolerance test (ITT) (q).
(r-s) Lipid droplet in high fructose-fed rat glomeruli detected by flow cytometry.
(t-u) Lipid droplet in fructose-exposed differentiated podocytes detected by flow cytometry.
(v-w) Serum TG and TC levels in high fructose-fed rats and normal rats.
(x-y) TG and TC levels in fructose-exposed differentiated podocytes.

**
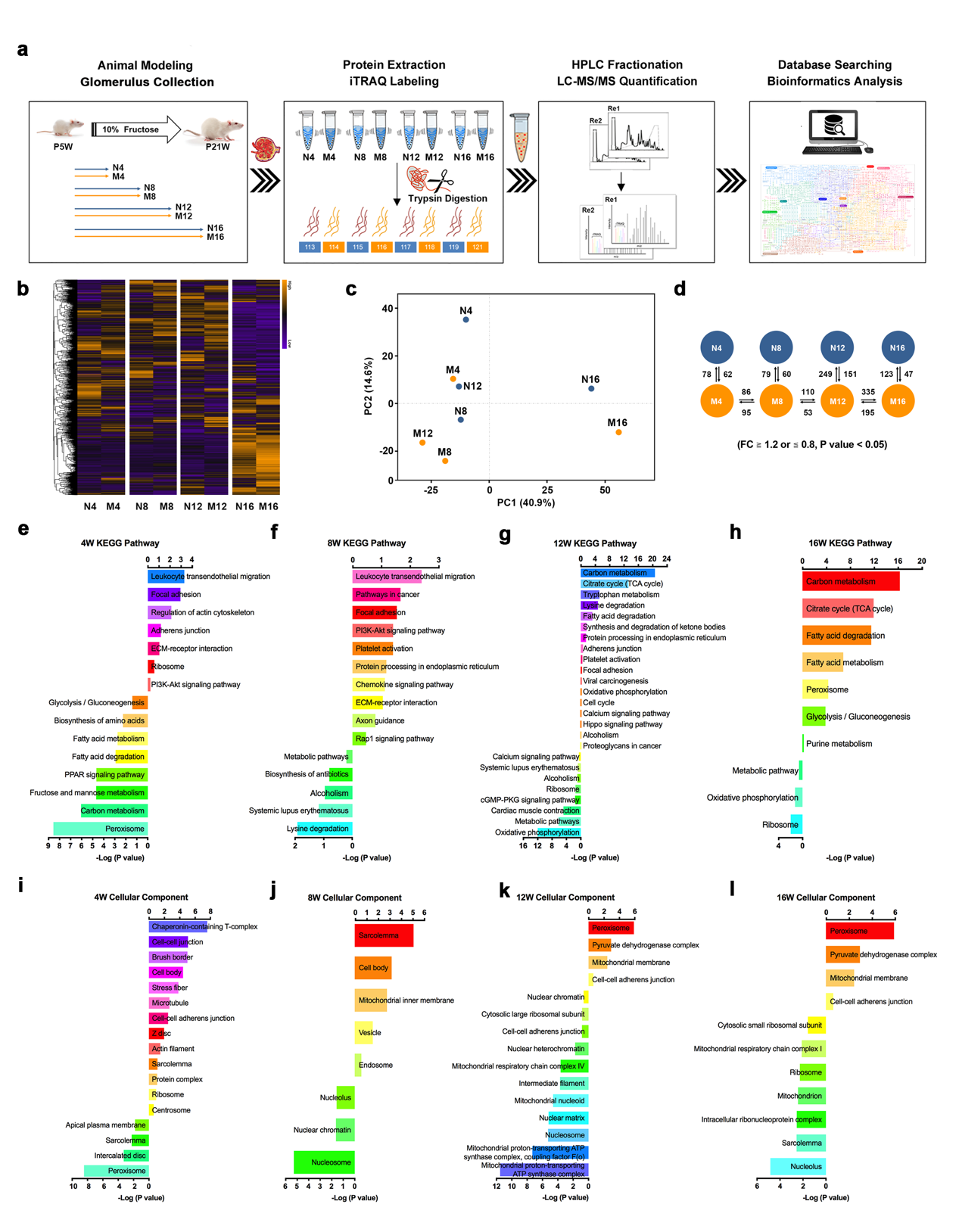
Figure S2. Quantitative proteomic profiling of dynamic changes in rat glomeruli induced by high fructose diet**

(a) Schematic workflow of quantitative proteomic profiling in glomeruli of high fructose-fed rats. M4 to M16 were rats fed by high fructose diet for 4, 8, 12 and 16 weeks, respectively, and N4 to N16 were correspondingly paired normal diet group.

(b-d) Overview of dynamic proteome changes in glomeruli of high fructose-fed rats. (b) Heatmap showed distinct protein expression pattern in normal and fructose-fed groups. (c) Principal component analysis showed N16 and M16 were deviated from the other six samples among eight N and M samples. (d) Significantly dysregulated proteins between each paired M vs. N group and at different fructose-modeling time points.

(e-h) KEGG pathway analysis of significantly dysregulated proteins at 4, 8, 12, and 16 weeks in high-fructose modeling groups compared to its corresponding normal diet groups.

(i-l) Cellular component analysis of significantly dysregulated proteins at 4, 8, 12, and 16 weeks in high-fructose modeling groups compared to its corresponding normal diet groups.

**
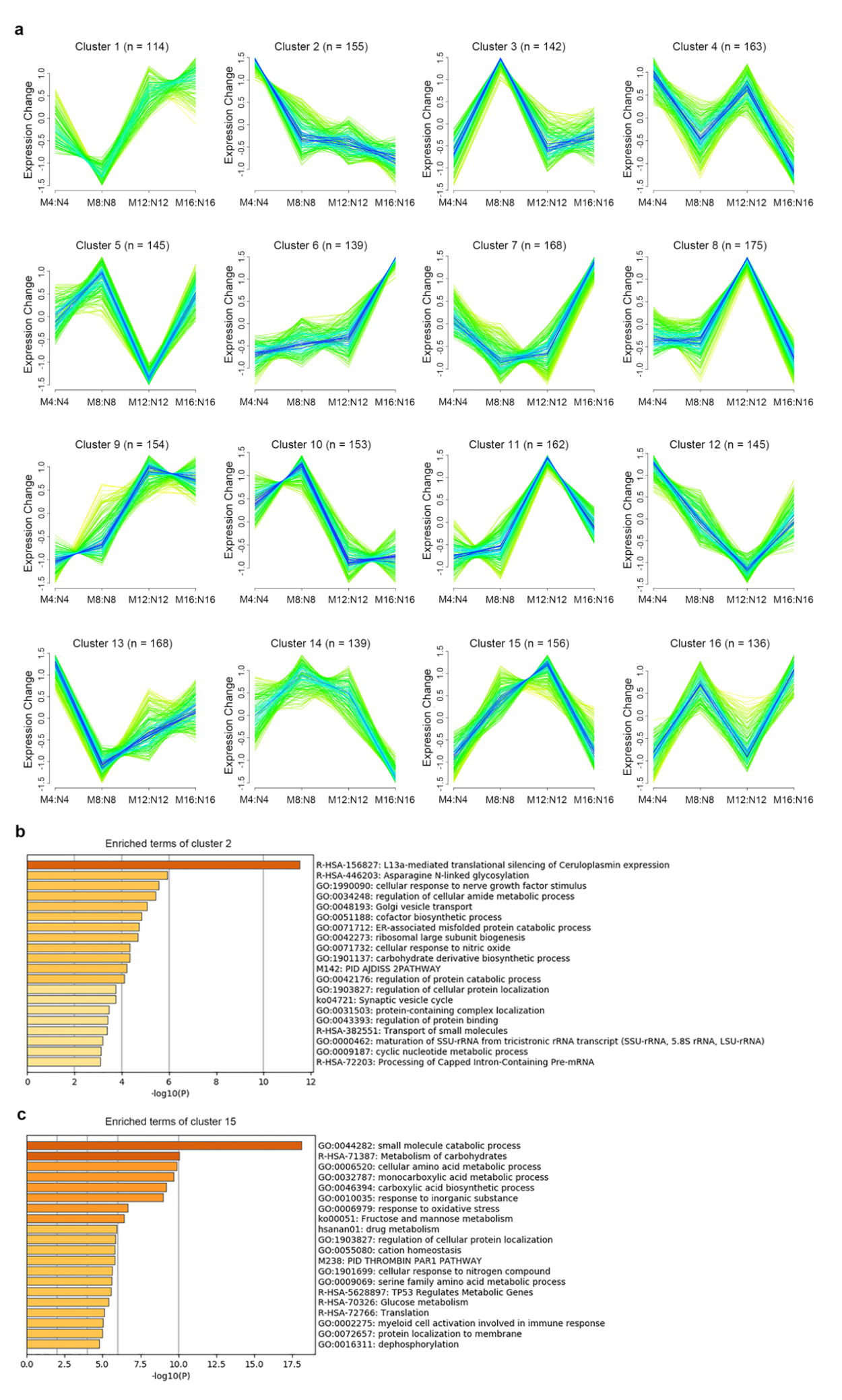
Figure S3. Soft clustering analysis of all quantified proteins in glomeruli of model group (M) vs. normal group (N)**

All quantified proteins were generally divided into 16 clusters with different changing trends (a), and the continuously down-regulated cluster 2, the continuously up-regulated cluster 6 (b) and cluster 15 with a sharp decrease at the 16^th^ week (c) were selected for detailed analysis.


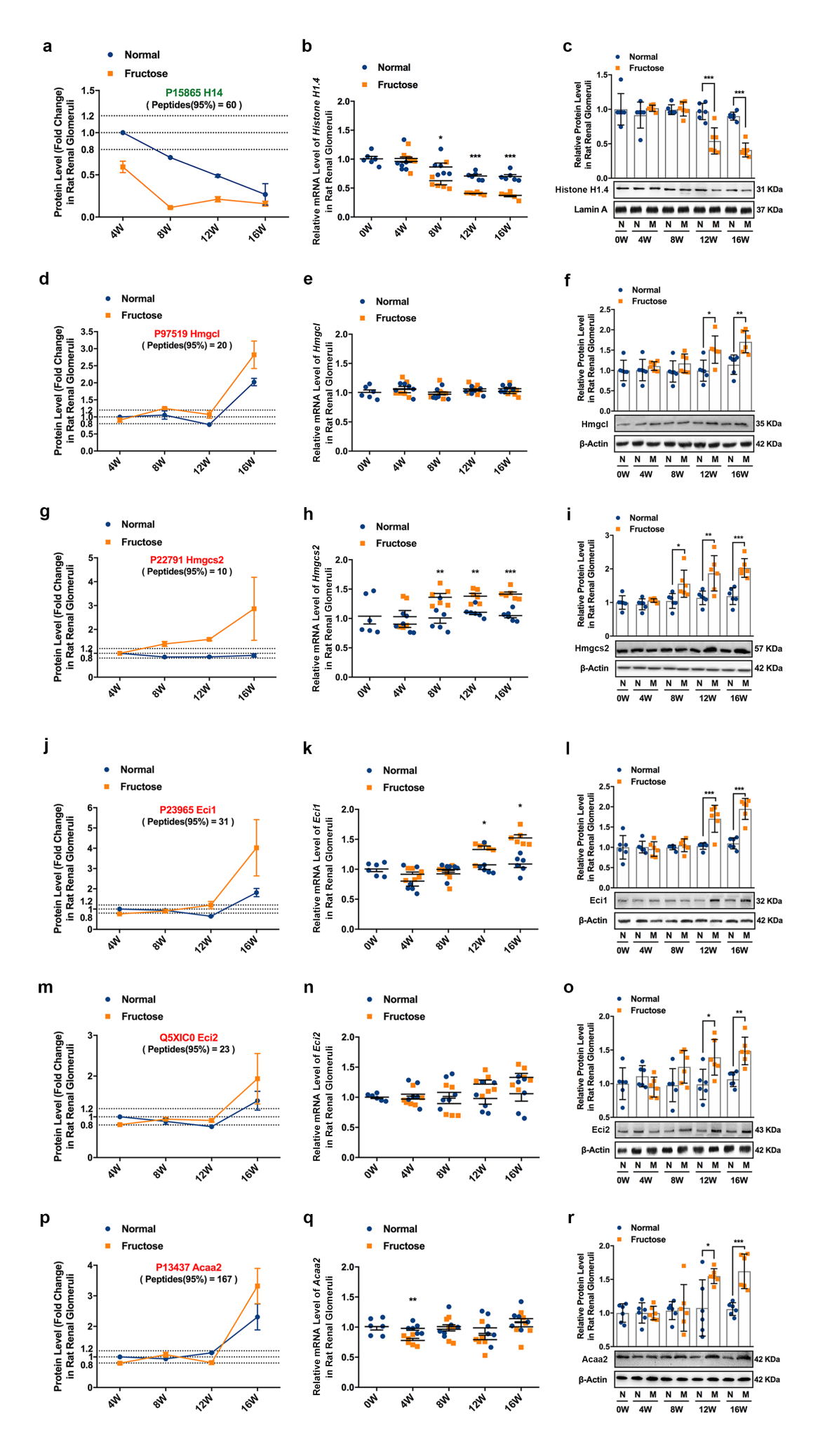


**Figure S4.Validation of quantitative proteomics data using real-time qPCR and Western blot**

The expression level of Histone H1.4 (a-c), Hmgcl (d-f), Hmgcs2 (g-i), Eci1 (j-l), Eci2 (m-o) and Acaa2 (p-r) in the glomeruli of high fructose-fed rats were quantified by iTRAQ, and validated by real-time qPCR (n=6) and Western Blot (n=6), respectively.

Relative protein or mRNA levels of Histone H1.4, Hmgcl, Hmgcs2, Eci1, Eci2 and Acaa2 were normalized to β-Actin, respectively. Mean values ± SD are shown. Student’s two-tailed paired *t* test was used for comparing two groups. One-way ANOVA with Tukey’s post hoc test was used for multi-group comparisons. **p* < 0.05, ***p* < 0.01 and ****p* < 0.001 denote the significant difference as compared to the corresponding normal animal group or normal cell group.


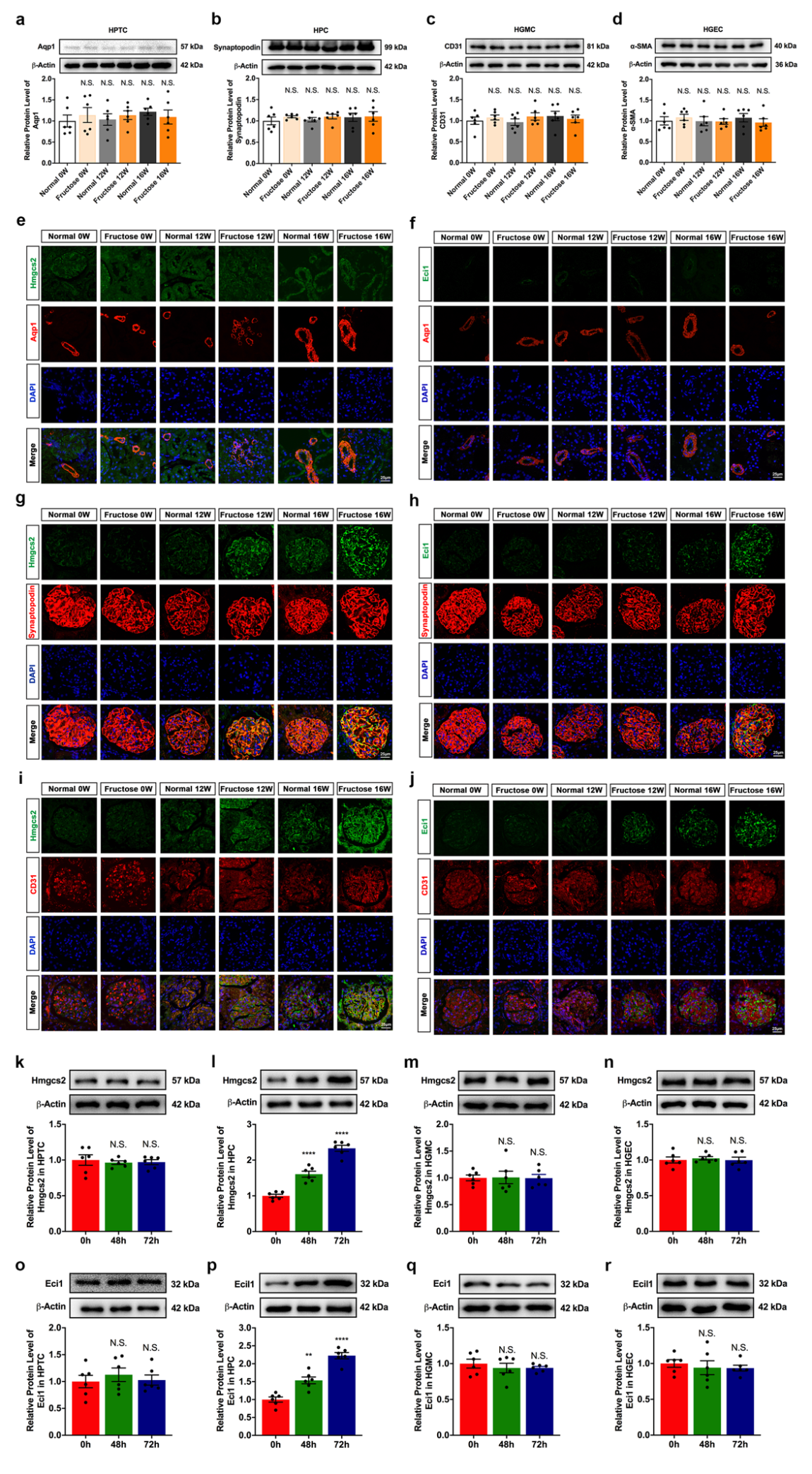


**Figure S5. Hmgcs2 and Eci1 were specifically up-regulated in podocytes**

(a-d) Relative protein levels of marker proteins of different renal cell types including HPTC from tubule and HPC, HGMC and HGEC from glomeruli in mesh-selected rat glomeruli. (a) Relative protein levels (normalized by β-Actin) of HPTC marker Aqp1; (b) Relative protein levels (normalized by β-Actin) of HPC marker Synaptopodin. (c) Relative protein levels (normalized by β-Actin) of HGMC marker CD31. (d) Relative protein levels (normalized by β-Actin) of HGEC marker α-SMA.

(e-j) Immunofluorescence of Hmgcs2 and Eci1 in different renal cell types including proximal tubular cells (e, f), podocytes (g, h), and mesangial cells (i, j) in kidney cortex of high fructose-fed rats.

(k-r) Relative protein levels of Hmgcs2 and Eci1 in response to fructose treatment in different renal cell types detected by Western blot. (k-n) Relative protein levels of Hmgcs2 (normalized by β-Actin) in HPTCs (human renal proximal tubular cells), HPCs (human renal podocytes), HGMCs (human renal mesangial cell) and HGECs (human renal microvascular endothelial cells) showed only Hmgcs2 in HPCs was specifically up-regulated in response to fructose treatment; (o-r) Relative protein levels of Eci1 (normalized by β-Actin) in HPTCs, HPCs, HGMCs and HGECs showed only Eci1 in HPCs was specifically up-regulated in response to fructose treatment.


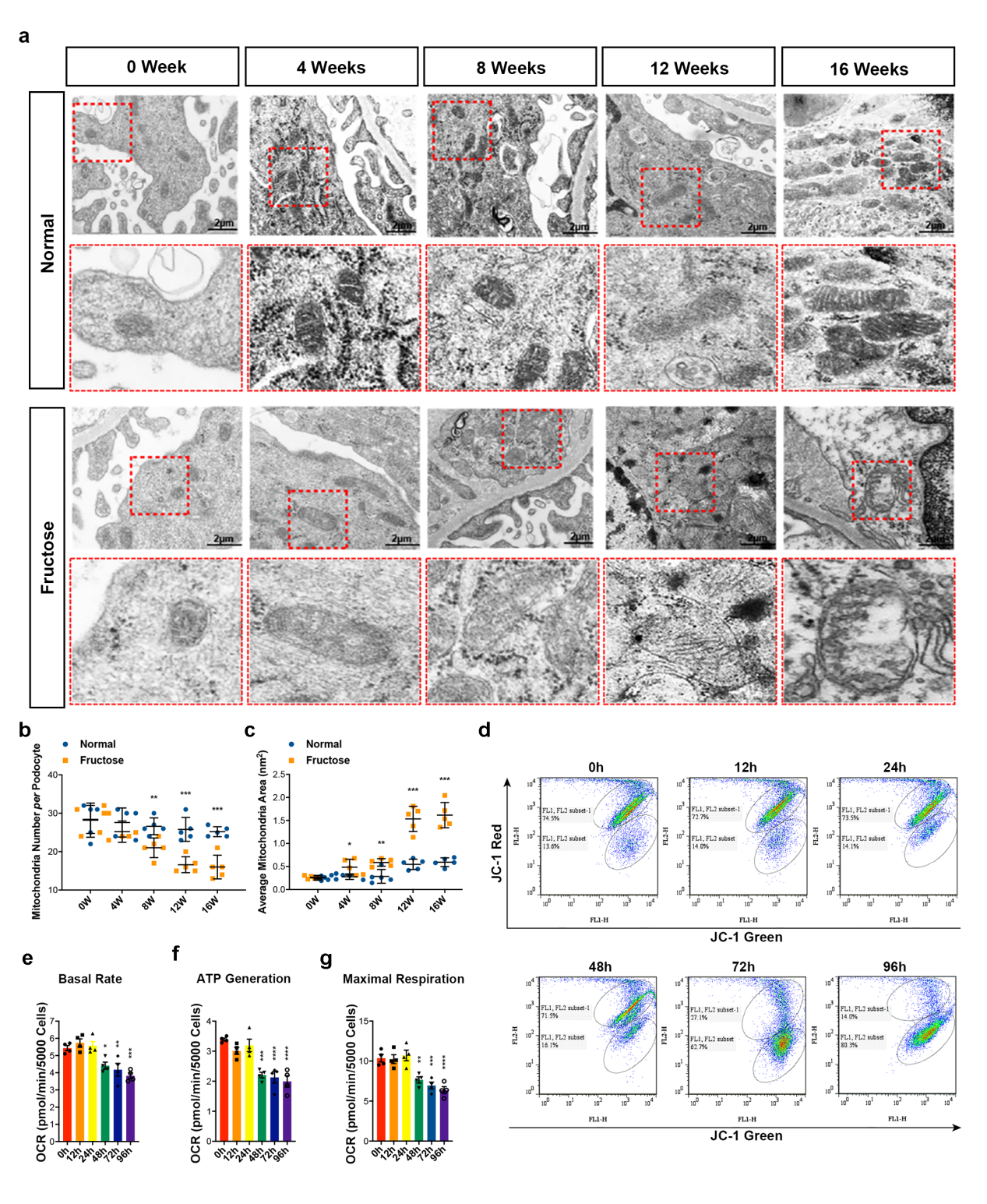


**Figure S6. High fructose induced abnormal mitochondria morphology and mitochondrial dysfunction in podocytes**

(a-c) The mitochondria ultrastructure was severely disrupted in glomerular podocytes of high fructose-fed rats. (a) Representative images of mitochondria ultrastructure by transmission electron microscopy. The total number of mitochondria (b) and the size (c) were quantitated in glomerular podocytes of normal and model groups (n=5 *per* group).

(d) The mitochondria depolarization and *ΔΨ*m reduction in fructose-exposed podocytes. *ΔΨ*m was detected in 5 mM fructose-exposed differentiated podocytes by flow cytometry analysis *via* JC-1 staining (n=5 *per* group).

(e-g) High fructose induced significant mitochondrial dysfunction in podocytes. Mito stress test was performed to measure the key parameters of mitochondrial function in differentiated podocytes with or without 5 mM fructose exposure using Seahorse × 96 Extracellular Flux Analyzer (Seahorse Bioscience). Statistics of key parameters of respiratory function including basal rate (e), ATP generation (f) and maximal respiration (g) in differentiated podocytes with or without 5 mM fructose exposure (n=10 *per* group).


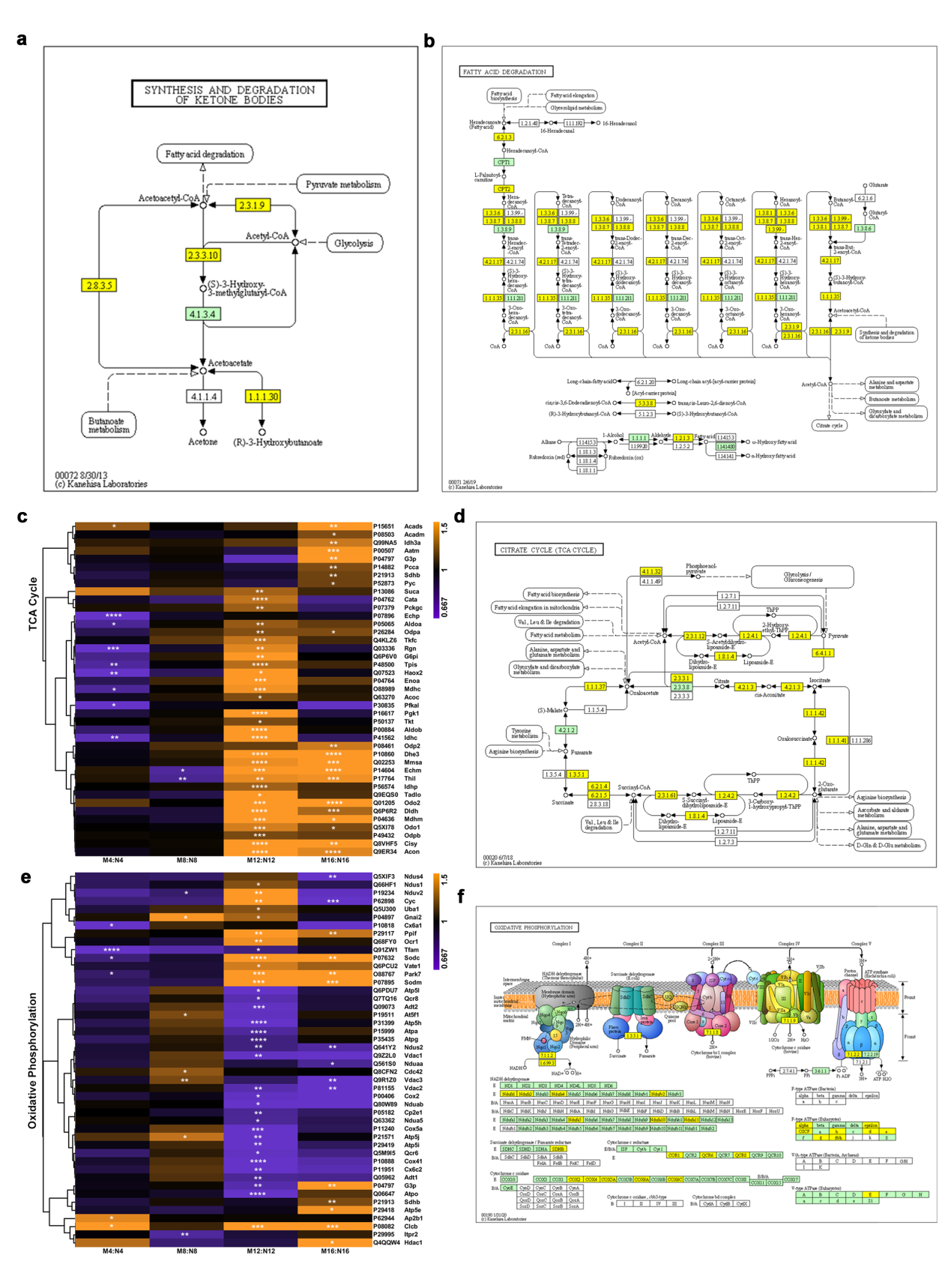


**Figure S7. Ketogenesis and fatty acid degradation were up-regulated in rat glomeruli by high fructose diet**

(a-b) KEGG pathway mapping of up-regulated proteins in ketogenesis (a) and fatty acid degradation pathway (b) during the whole fructose-modeling period. Nodes highlighted in light green indicated proteins or enzymes existing in the pathway in rats (*Rattus norvegicus*). Nodes highlighted in yellow indicated proteins or enzymes remarkably dysregulated in high fructose-fed group compared with the normal group.

(c-d) Up-regulation of TCA cycle as displayed by heatmap (c) and KEGG map (d).

(e-f) Dysregulation of oxidative phosphorylation as displayed by heatmap (e) and KEGG map (f).


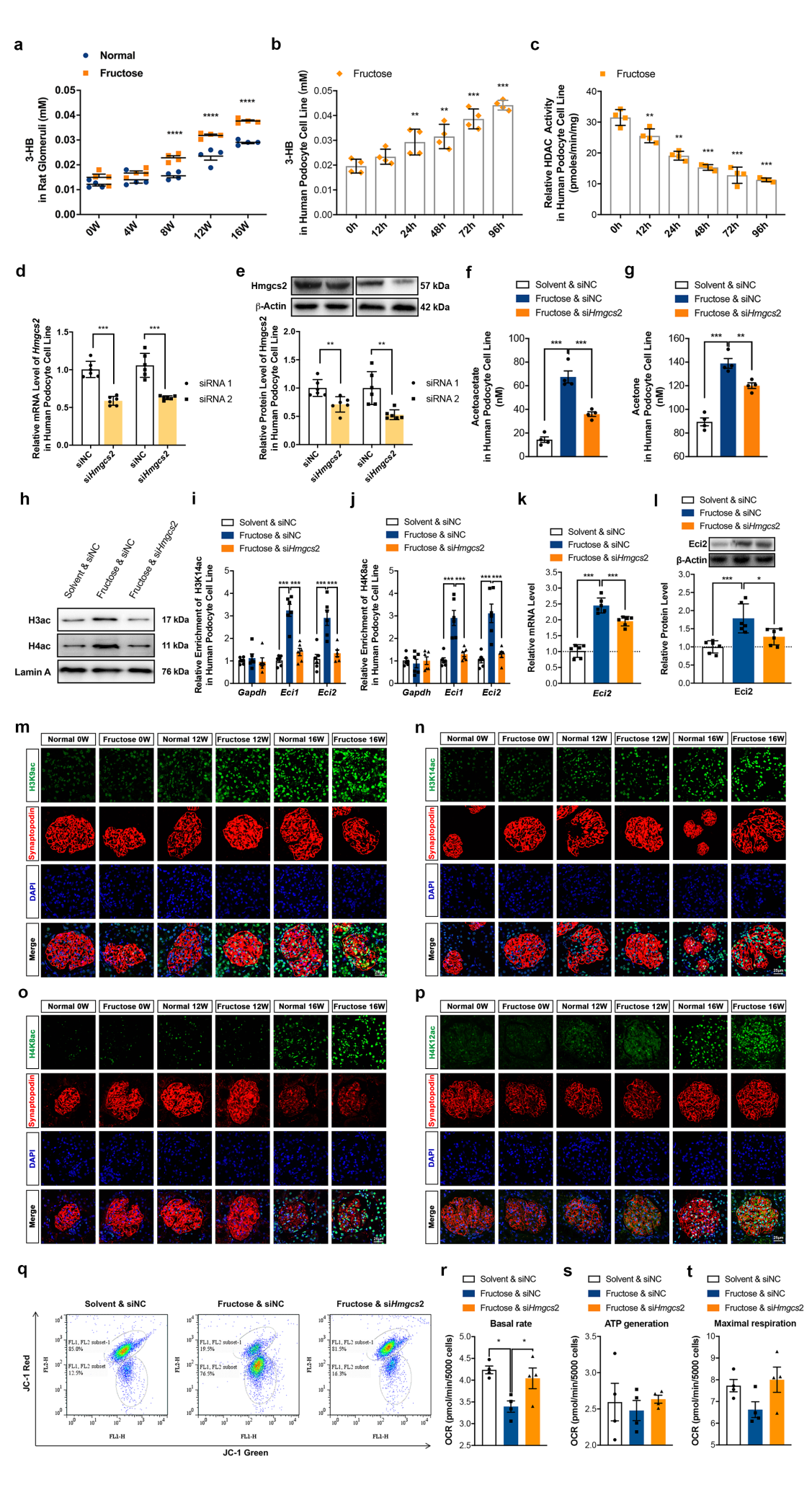


**Figure S8. Key enzymes in fatty acid degradation were up-regulated by high fructose-induced HDACs inhibition and histone hyper-acetylation *via* enhanced ketone body level**

(a-b) Time-dependent increase of ketone body 3-HB in glomeruli of high fructose-fed mice (a) and high fructose-exposed differentiated podocytes (b) (n=4 *per* group).

(c) HDACs activity was time-dependently inhibited by fructose exposure in differentiated podocytes (n=4*per* group).

(d-e) Knockdown of *Hmgcs2* significantly reduced Hmgcs2 expression at both mRNA (d) and protein (e) levels.

(f-g) Knockdown of *Hmgcs2* significantly reduced ketone bodies levels of AcAc (f) and acetone (g) induced by high fructose in differentiated podocytes.

(h) Total H3ac (histone H3 acetylation) and H4ac were enhanced in differentiated podocytes by high fructose exposure, but reversed by knockdown of *Hmgcs2* prior to fructose exposure. Differentiated podocytes were transfected with *Hmgcs2* siRNA as well as the negative control (siNC), and then cultured with or without 5 mM fructose (n=6 *per* group).

(i-j) The increasing levels of H3K14ac (i) and H4K8ac (j) at *Eci1* or *Eci2* promoter induced by high fructose in differentiated podocytes were significantly reversed by knockdown of *Hmgcs*2. Enrichment of H3K14ac and H4K8ac at *Eci1* or *Eci2* promoter were measured by real-time qPCR and quantitated in differentiated podocytes transfected with *Hmgcs2*siRNA as well as siNC, then cultured with or without 5 mM fructose, respectively. *Gapdh* was used as negative control (n=6*per* group).

(k-l) Knockdown of *Hmgcs2* reversed the increase of Eci2 induced by high fructose in differentiated podocytes as measured by real-time qPCR (k) and Western blot (l) (n=6 *per* group), respectively. Relative mRNA or protein levels of Eci2 were normalized to β-Actin, respectively.

(m-p) Immunofluorescence showed increase of H3K9ac (m), H3K14ac (n), H4K8ac (o) and H4K12ac (p) at 12^th^ and 16^th^ week in kidney glomeruli of high fructose-fed rats.

(q) Knockdown of *Hmgcs2* efficiently rescued the decrease of mitochondrial membrane potential induced by high fructose in differentiated podocytes. Flow cytometry analysis of *△Ψm* was performed in differentiated podocytes transfected with *Hmgcs2* siRNA as well as siNC, and then cultured with or without 5 mM fructose (n=5 *per* group).

(r-t) Knockdown of *Hmgcs2* reversed the inhibitory effects of high fructose on basal rate, ATP generation and maximal respiration in differentiated podocytes. Bioenergetics profile was measured by OCR with a Seahorse × 96 Extracellular Flux Analyzer (Seahorse Bioscience) in differentiated podocytes. Statistics of key parameters of respiratory function including basal rate (r), ATP generation (s) and maximal respiration (t) in differentiated podocytes transfected with *Hmgcs2* siRNA as well as siNC, and then cultured with or without 5 mM fructose (n=6 *per* group).


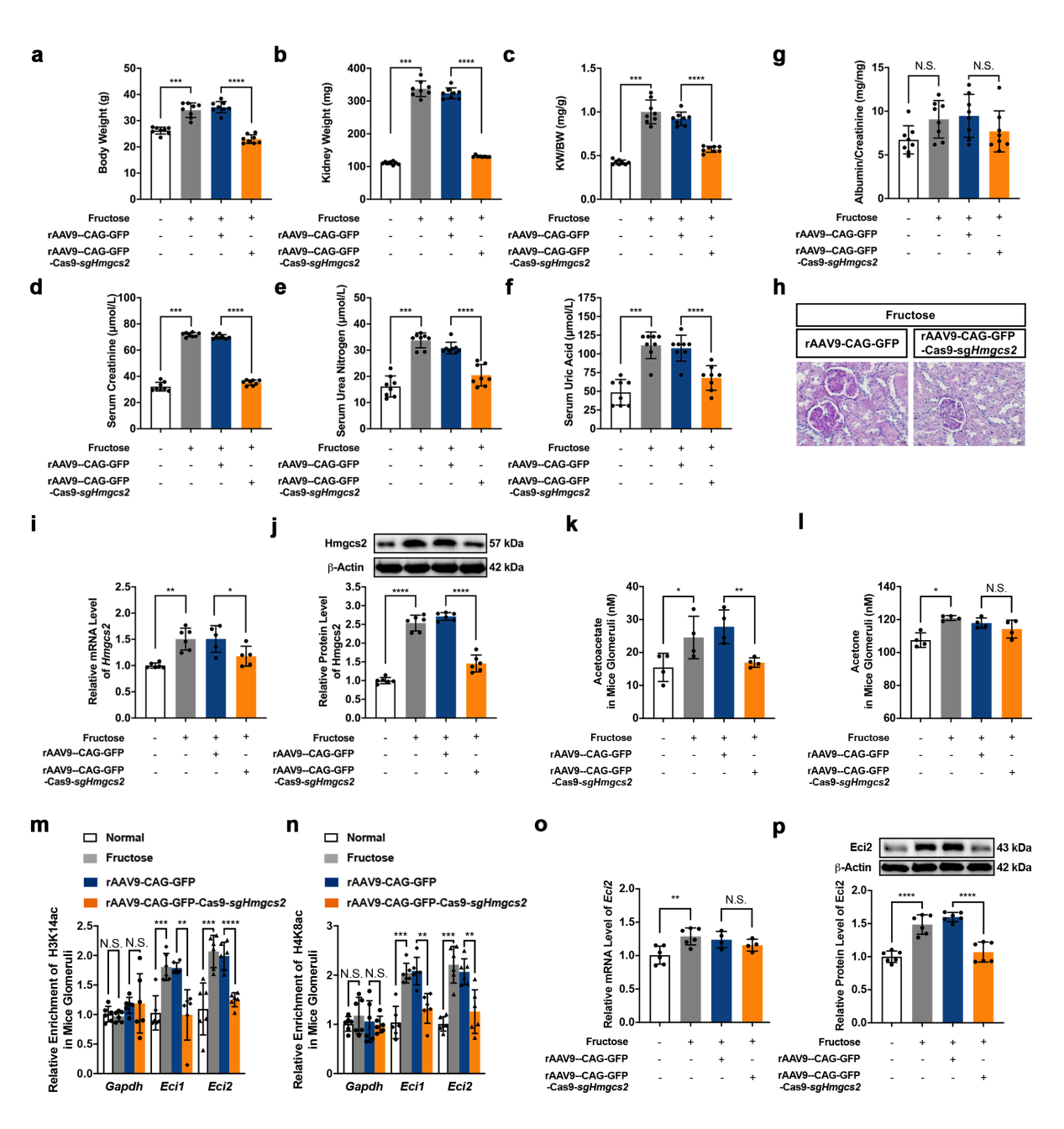


**Figure S9. High fructose-induced mitochondrial dysfunction in podocytes can be reversed by knockdown of key enzymes in ketogenesis**

(a-h) Phenotype of kidney-specific *Hmgcs2* knockdown mice.(a-g) Physiological and biochemical phenotypes including body weight (BW, a), kidney weight (KW, b), KW to BW ratio (KW/BW, c), creatinine (d), urea nitrogen (e) and uric acid (f) levels in serum, and albumin to creatinine ratio (g). (h) Periodic acid–Schiff (PAS) staining of mouse glomeruli.

(i-j) Relative mRNA (i) and protein (j) levels of Hmgcs2 in glomeruli of kidney-specific *Hmgcs2* knockdown mice.

(k-l) Ketone bodies levels of AcAc and acetone in glomeruli of kidney-specific *Hmgcs2* knockdown mice.

(m-n) The increasing levels of H3K14ac (m) and H4K8ac (n) at *Eci1* or *Eci2* promoter induced by high fructose in mouse glomeruli were significantly reversed by kidney-specific *Hmgcs2* knockdown. Enrichment of H3K9ac and H4K12ac at *Eci1* or *Eci2* promoter were measured by real-time qPCR and quantitated in mouse glomeruli under indicated conditions. *Gapdh* was used as negative control (n=6 *per* group).

(o-p) Knockdown of *Hmgcs2* reversed the increase of Eci2 in glomeruli of high fructose-fed mice as measured by real-time qPCR (o) and Western blot (p) (n=6 *per* group), respectively. Relative mRNA or protein levels of *Eci2* were normalized to *β-Actin*, respectively.

**
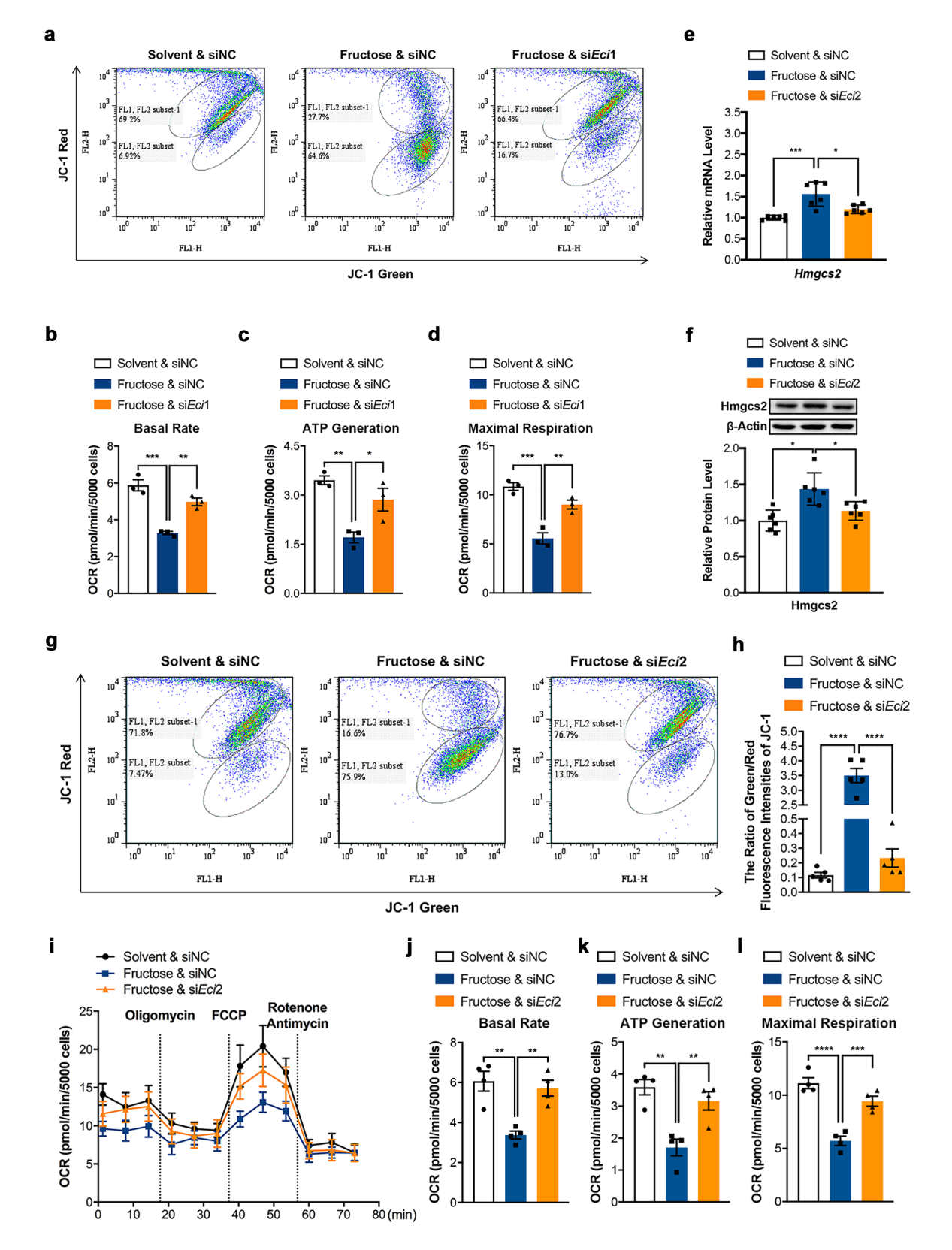
**

**Figure S10. High fructose-induced mitochondrial dysfunction in differentiated podocytes can be reversed by knockdown of *Eci1* or *Eci2* in fatty acid degradation**

(a) Knockdown of *Eci1* efficiently rescued the decrease of mitochondrial membrane potential induced by high fructose in differentiated podocytes. Flow cytometry analysis of *△Ψm* was measured in differentiated podocytes transfected with *Eci1* siRNA as well as siNC, and then cultured with or without 5 mM fructose (n=5 *per* group).

(b-d) Knockdown of *Eci1* reversed the inhibitory effects of high fructose on basal rate, ATP generation and maximal respiration in differentiated podocytes. Bioenergetics profile was measured by OCR with a Seahorse × 96 Extracellular Flux Analyzer (Seahorse Bioscience) in differentiated podocytes. Statistics of key parameters of respiratory function including basal rate (b), ATP generation (c) and maximal respiration (d) in differentiated podocytes transfected with *Eci1* siRNA as well as siNC, and then cultured with or without 5 mM fructose (n=6 *per* group).

(e-f) Knockdown of *Eci2* reversed the increase of Hmgcs2 induced by high fructose in differentiated podocytes. mRNA (e) and protein (f) levels of Hmgcs2 were measured in differentiated podocytes transfected with *Eci2* siRNA as well as siNC, and then cultured with or without 5 mM fructose (n=6 *per* group). Relative mRNA or protein levels of Hmgcs2 were normalized to β-Actin, respectively.

(g-h) Knockdown of *Eci2* efficiently rescued the decrease of mitochondrial membrane potential induced by high fructose in differentiated podocytes. Flow cytometry analysis of *△Ψm* was performed in differentiated podocytes transfected with *Eci2* siRNA (g) as well as siNC, and then cultured with or without 5 mM fructose. (h) The corresponding quantification results of g (n=5 *per* group), respectively.

(i-l) Knockdown of *Eci2* reversed the inhibitory effects of high fructose on basal rate, ATP generation and maximal respiration in differentiated podocytes. Bioenergetics profile was measured by OCR with a Seahorse × 96 Extracellular Flux Analyzer (Seahorse Bioscience) in differentiated podocytes transfected with *Eci2* siRNA as well as the respective negative control (siNC), and then cultured with or without 5 mM fructose (n=6 *per* group) (i). Oligomycin (1 mM), Carbonyl cyanide 4-(trifluoromethoxy) phenylhydrazone (FCCP) (1 mM), and rotenone (0.5 mM)/antimycin A (0.5 mM) were added at the times indicated. Statistics of key parameters of respiratory function including basal rate (j), ATP generation (k) and maximal respiration (l) in differentiated podocytes transfected with *Eci1* or *Eci2* siRNA as well as siNC, and then cultured with or without 5 mM fructose (n=6 *per* group).

**Table S1. Information of protein samples for iTRAQ labeling**

| **iTRAQ Labeling** | **Biological Replicates** | |
| --- | --- | --- |
|  | **Re1** | **Re1** |
| 113 | N4-1 | N4-2 |
| 114 | M4-1 | M4-2 |
| 115 | N8-1 | N8-2 |
| 116 | M8-1 | M8-2 |
| 117 | N12-1 | N12-2 |
| 118 | M12-1 | M12-2 |
| 119 | N16-1 | N16-2 |
| 121 | M16-1 | M16-2 |

* N: normal group; M: fructose modeling group;

* 4, 8, 12, 16: fructose modeling time (weeks).

The rats in fructose modeling groups at 4^th^, 8^th^, 12^th^ , and 16^th^ week were indicated as M4, M8, M12, and M16, whereas their corresponding normal groups with regular diet were indicated as N4, N8, N12, and N16, respectively.

**Table S2. siRNA sequences for selected genes**

| **Symbol** | **Sense primer (5’→3’)** | **Antisense primer (5’→3’)** |
| --- | --- | --- |
| *Eci1*siRNA 1 | CCAAGGCCAUGAUGCGAAATT | UUUCGCAUCAUGGCCUUGGTT |
| *Eci1* siRNA 2 | CCUGCAGAUGUACUUAGAGTT | CUCUAAGUACAUCUGCAGGTT |
| *Eci2* siRNA 1 | GGAAUGAUCUGACUAACUUTT | AAGUUAGUCAGAUCAUUCCTT |
| *Eci2* siRNA 2 | GCAACAUUUCAUACACCAUTT | AUGGUGUAUGAAAUGUUGCTT |
| *Hmgcs2*siRNA 1 | CCUGGAGAAGUAUAACAAUTT | AUUGUUAUACUUCUCCAGGTT |
| *Hmgcs2* siRNA 2 | GGGAUGGUCGUUAUGCCAUTT | AUGGCAUAACGACCAUCCCTT |
| Negative control | UUCUCCGAACGUGUCACGUTT | ACGUGACACGUUCGGAGAATT |

**Table S3. Primer sequences of selected genes for real-time qPCR**

| **Symbol** | **Sense primer (5’→3’)** | **Antisense primer (5’→3’)** |
| --- | --- | --- |
| *Eci1* | CCGCCATTATCCTGTAGTCA | GCATCCGAGGTGTCATCC |
| *Eci2*  *Acaa2* | CATAGACGGCATCAAATAG  CTGCTCCGAGGTGTGTTTGTA | TGGGAATGACCTGACTAAC  GGCAGCAAATTCAGACAAGTCA |
| *Hmgcl* | TGTTCAGGGCTTGACAGAT | ATGGGAGTGAGCGTTGTG |
| *Hmgcs2* | CAGGCGTTGGTGGTATCT | GTATGGGCTTCTGTTCGG |
| *Histone H1.4* | AGGCAAAGGCAACTAAGGCTA | CTTTAGGCTTTACCGTTTTCGC |
| *β-Actin* | GTGACGTTGACATCCGTAAAGA | GCCGGACTCATCGTACTCC |
